# A Spontaneous Isolated Superior Mesenteric Artery Dissection Associated with Cocaine Abuse: A Pathomechanistic Association

**DOI:** 10.1155/2020/2514687

**Published:** 2020-06-06

**Authors:** Rayan S. El-Zein, Jeffrey Sobecki, Roy Greenberg, Michael Keleher, Robert A. Palma

**Affiliations:** ^1^Department of Internal Medicine, OhioHealth Doctors Hospital, 5100 W Broad St, Columbus, OH 43228, USA; ^2^Department of Interventional Radiology, OhioHealth Doctors Hospital, 5100 W Broad St, Columbus, OH 43228, USA

## Abstract

Spontaneous isolated superior mesenteric artery dissection (SISMAD) is a rare potentially fatal disease. We present a case of cocaine-related SISMAD in a patient with abdominal pain. A 38-year-old African American male with hypertension and alcohol, cocaine, and tobacco abuse presented with abdominal pain and recent cocaine use. A CT angiogram revealed SISMAD; he was treated with conservative management. Cocaine and SISMAD share similar pathophysiologic mechanisms pertaining to vascular smooth muscle cell apoptosis and increased shear stress at fixed vascular positions. Our report emphasizes the need to consider cocaine abuse in SISMAD pathophysiology, risk stratification, and treatment algorithms in future studies.

## 1. Introduction

Initially reported by Bauersfeld in 1947, spontaneous isolated superior mesenteric artery dissection (SISMAD) is a rare but potentially fatal arterial disease with an incidence of 0.06% from a cohort of 6666 autopsies [1]. Prior to 2001, only 46 cases were reported; since 2016, more than 622 cases have been reported [2] reflecting the advancement of imaging rather than prevalence. Multiple risk factors have been suggested such as arteriopathy (connective tissue diseases, cystic medial necrosis, etc.), tobacco use, atherosclerosis, alcohol abuse, obesity, heavy weight lifting, and pregnancy [3]. While cocaine use has been associated with other mesenteric pathology (ischemia, perforation), cocaine-related SISMAD remains a rare finding.

## 2. Case Report

A 38-year-old African American male with a history of hypertension and alcohol, cocaine, and tobacco use presented with recurrent abdominal pain. Three days prior, he was admitted with abdominal pain and hypertensive urgency. Cocaine and cannabinoids were found in his urine. A computed tomography (CT) scan of the abdomen with IV contrast demonstrated atherosclerosis of the aortoiliac system. With conservative management, the patient was diagnosed with cocaine-induced vasospasm and was discharged after two days.

The following day, he returned with recurrence of his abdominal pain and reported continued cocaine use. He described his symptoms as crampy in nature localizing to the upper right and left quadrants. He denied any associated nausea, vomiting, diarrhea, melena, or hematochezia. On arrival, his blood pressure was 190/120 mmHg. His abdomen was soft and flat with diffuse abdominal tenderness to soft and deep palpation with no guarding, organomegaly, rebound tenderness, or peritoneal signs.

**Figure 1:**
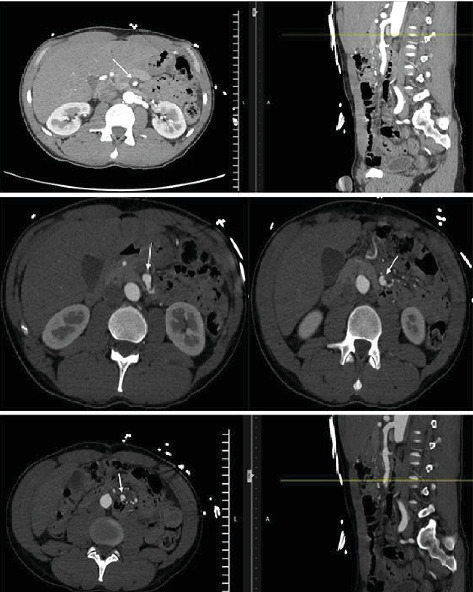
SISMAD within the proximal-to-mid SMA with a 3 cm segment along the proximal SMA with thrombosis of the false lumen extending craniocaudally; 70% stenosis of the proximal SMA; an intimal flap was seen intermittently below this level extending to the major bifurcations of the middle colic artery.

Results of laboratory tests revealed a serum lactic acid of 2.7 mmol/L. A CT angiogram (CTA) of the abdomen and pelvis revealed a SISMAD within the proximal-to-mid SMA with a 3 cm thrombosis of the proximal false lumen and distal false lumen patency; 70% stenosis of the proximal SMA was noted secondary to this process (Figure 1). There was no evidence of abdominal ischemia. He was admitted to a medical/surgical floor with telemetry and was treated with conservative management. In addition to pain control and bowel rest, his home amlodipine 5 mg daily and hydrochlorothiazide 12.5 mg daily were restarted with the addition of as-needed hydralazine for a strict systolic blood pressure target of <140. In conjunction with the vascular surgery team, he was started on antiplatelet monotherapy with aspirin 325 mg daily. Two days after presentation, a repeat CT angiogram showed stabilization of the SMA dissection and the patient was safely discharged. The patient did not have any further abdominal imaging after discharge.

## 3. Discussion

SISMAD is rare with <1000 reported cases [2, 3]. Clinical presentations can range from an asymptomatic incidental finding to severe abdominal pain from bowel ischemia or fatal aneurysmal rupture. The severity of the pain has been shown to correlate with the length of dissection [4]. CTA is considered the gold standard [5].

Five classifications have been proposed by Sakamoto et al., Yun et al., Zerbib et al., Luan et al., and Li et al. [6]. Yun's classification based on dissection entry and reentry points, length, patency, and degree of luminal stenosis has become the most commonly used due to its simplicity [4]. The SISMAD in our case is classified as a combination of type 2a and type 2b. The proximal false lumen is thrombosed; however, there is a visible false lumen distal to thrombosis without an obvious reentry site. Previously, Park et al. published a series in which they found that Yun type 2 (2a or 2b) dissections were more likely to undergo complete remodeling [7].

Although the precise pathogenesis of SISMAD remains uncharacterized, it is suggested to be related to arterial wall dysfunction involving atherosclerosis, vasculitis, cystic medial necrosis, segmental arteriolar mediolysis, fibromuscular dysplasia, and connective tissue diseases [3, 8, 9]. Histopathological and hemodynamic components have also been established which point towards a relationship with cocaine. Cocaine is associated with several arterial dissections: coronary [10], aortic [11], carotid [12], and renal artery dissections [13]. The sympathomimetic action of cocaine leads to significantly increased shear stress resulting in intimal tear, especially in fixed vascular positions (e.g., ligamentum arteriosum) as seen in aortic dissections [11]. Approximately 1-3 cm from the SMA ostium, the SMA transitions from a fixed retropancreatic segment to an unfixed mesenteric segment where most SISMADs originate. Flow dynamic studies revealed greater shear stress at this point along the anterior convex portion of the SMA [14, 15]. In a study by Bigi et al. [16], chronic cocaine use (average 10 years) was associated with impairment of elastic properties of the aorta; another study on cultured rat aortic vascular smooth muscle cells (VSMCs) demonstrated that aortic VSMCs undergo rapid apoptosis in response to cocaine [17]. Concurrently in SISMAD, elastic fiber fragmentation and loss of smooth muscle cells are seen histologically thus reflecting increased wall weakness and decreased distensibility [3].

In our case, we postulated that our patient's chronic cocaine abuse along with other risk factors (atherosclerosis, tobacco, and alcohol) has likely resulted in gradual SMA wall dysfunction forming a nidus for subsequent dissection. His recent acute cocaine use may have produced a significant shear stress elevation leading to dissection. Therefore, with the aforementioned mechanisms, our patient's cocaine use played a significant role in his disease development.

No prospectively randomized clinical trial has been published regarding treatment guidelines of SISMAD. Treatment options range from conservative management with or without antithrombotic therapy (anticoagulation or antiplatelet), endovascular SMA stenting, or open surgery. In a recent meta-analysis of 25 selected studies, conservative medical treatment was successful in 397 of 447 of cases, irrespective of anticoagulation or antiplatelet therapy, and 50 underwent conversion to endovascular therapy or open surgery [18]. Park et al. showed that 41% treated conservatively had improvement on surveillance CTA and 15% of patients had complete remodeling [7]. There has been no difference in outcomes with the use of anticoagulants or antiplatelet agents, as anticoagulation may prevent false lumen thrombosis and promote dissection propagation [4]. Persistent abdominal pain or significant stenosis (>80%) indicates endovascular stenting whereas hemorrhage or bowel ischemia necessitates open surgery [3]. It is reasonable to apply current recommendations for patients with SISMAD unrelated to cocaine to patients with cocaine-related SISMAD. This report emphasizes the need to consider cocaine in SISMAD risk stratification, pathophysiology, and treatment algorithms in future studies.
